# Retrospective Analysis of Rituximab Therapy for Myasthenia Gravis: A Case Series

**DOI:** 10.7759/cureus.74372

**Published:** 2024-11-24

**Authors:** Abeer S Albalawi, Mohammed Alharbi, Thamer S Albalawi

**Affiliations:** 1 Neuroscience Department, King Abdullah Medical City, Makkah, SAU; 2 Laboratory Department, King Fahad Specialist Hospital, Tabouk, SAU

**Keywords:** acetylcholine receptor, mg-qol15, myasthenia gravis, neuromuscular junction disorder, rituximab

## Abstract

Myasthenia gravis (MG) is a neuromuscular junction disorder that involves several dysfunctions that eventually lead to muscle fatigue and weakness. Although immunotherapeutics are considered an effective treatment option for MG, treatment-refractory cases are documented. In this case report, we evaluate the efficacy and safety of rituximab in treating three cases of myasthenia gravis admitted to the neurology department of a tertiary hospital. We conducted a retrospective analysis on three cases of MG treated with rituximab at the neurology department of King Abdullah Medical Hospital. Rituximab was used as a first-line treatment in three patients with new-onset, non-refractory generalized MG who tested positive for acetylcholine receptor antibodies and failed to respond adequately to conventional therapies. Changes in disease severity and quality of life were assessed using the 15-item Myasthenia Gravis Quality of Life (MG-QOL15) scale. We observed a significant improvement in disease symptoms and quality of life in all patients.

Additionally, the oral prednisolone dose was reduced to less than 15 mg for all patients, with no reported side effects. This study suggests that patients with MG who test positive for acetylcholine receptor antibodies may benefit from rituximab as a safe and effective treatment alternative. In the three cases included in this analysis, rituximab led to notable improvements in quality of life, general clinical condition, and muscle weakness. Further studies with larger patient populations and longer follow-up periods are needed to validate these results and provide a more comprehensive understanding of rituximab's role in MG treatment.

## Introduction

Myasthenia gravis (MG) is the most prevalent neuromuscular junction disorder, characterized by muscle fatigue and weakness. MG is a chronic autoimmune disease with an annual incidence rate of one to two cases per 100,000 people and a prevalence of 20-50 cases per 100,000 people [1]. MG can be diagnosed through serological tests, electrophysiological studies, and clinical assessments.

Most patients with MG have detectable pathogenic autoantibodies against neuromuscular junction structures [2,3]. Various tests are readily available to evaluate synaptic dysfunction, and the pathophysiology of decreased neuromuscular transmission has been thoroughly investigated [4,5]. Most patients with MG develop autoantibodies against the acetylcholine receptor (AChR-Ab), muscle-specific kinase (MuSK-Ab), or agrin located on the postsynaptic membrane of the neuromuscular junction [6]. However, specific antibody profiles have not been detected in some MG cases. Fortunately, novel cell-based assays can detect the presence of AChR-Ab in such patients [7,8].

In neurology, treating MG presents several challenges [9]. One challenge is finding effective therapies that target the disease's underlying mechanisms while minimizing side effects. Currently, immunomodulatory agents such as corticosteroids, rituximab, mycophenolate mofetil, intravenous immunoglobulin (IVIg), and rapamycin are commonly used in the treatment of MG [10]. However, approximately 20% of patients with MG become refractory to these traditional treatments, leading to insufficient therapeutic responses, increased demand for plasma exchange, or repeated administration of IVIg infusions [10,11].

The issue of patients becoming refractory to MG treatment increases hospitalization rates and compromises the quality of life (QOL) for those affected [11]. MG affects QOL based on disease severity, which can eventually necessitate emergent interventions, such as mechanical ventilation [12]. Therefore, an alternative, safe, and effective therapy for refractory cases is essential to improving QOL and reducing hospitalizations. From this perspective, rituximab (RTX), a novel and promising monoclonal antibody, has been used over the past decade to treat MG.

RTX is a genetically engineered chimeric monoclonal antibody containing murine and human constant region sequences [13]. It targets the CD20 antigen present in B cells and has shown promise in the management of MG [14]. Also, RTX plays a crucial role in treating autoimmune disorders such as autoimmune hemolytic anemia, systemic lupus erythematosus (SLE), immune thrombocytopenic purpura (ITP), etc [15]. Unlike conventional cytotoxic chemotherapy and radiation therapy, targeted therapies like RTX focus on specific receptors or signaling events involved in disease progression, reducing toxicity to normal cells and potentially improving efficacy [14].

In this retrospective analysis, we evaluated the efficacy and safety of RTX as a first-line therapy to treat three non-refractory MG cases admitted to the neurology department of our tertiary hospital. This study is the first study in Saudi Arabia that used RTX as a first-line non-corticosteroid immunosuppressive treatment for non-refractory MG patients with anti-AChR autoantibody. We assessed the effect of RTX on disease progression, symptoms, and QOL in these patients using the Myasthenia Gravis Quality of Life 15 (MG-QOL-15) scale [16]. This scale consists of 15 questions reflecting the patient's well-being, with each question scored from 0 to 4, resulting in a total score ranging from 0-60 [17]. A higher score indicates a stronger impact of the disease on QOL [16]. Since only patients whose native language is Arabic or English are included in this investigation, the MG-QOL-15 English version that was used in this study was translated into Arabic. To guarantee accuracy, the translation was completed by a Saudi Arabian translation center with certification.

Our findings demonstrate that RTX therapy is effective in improving the clinical outcomes of patients with MG. The use of RTX reduces disease severity, decreases muscle weakness, and improves QOL. Furthermore, the therapy was well tolerated, with minimal adverse effects.

## Case presentation

Case one

**Figure 1:**
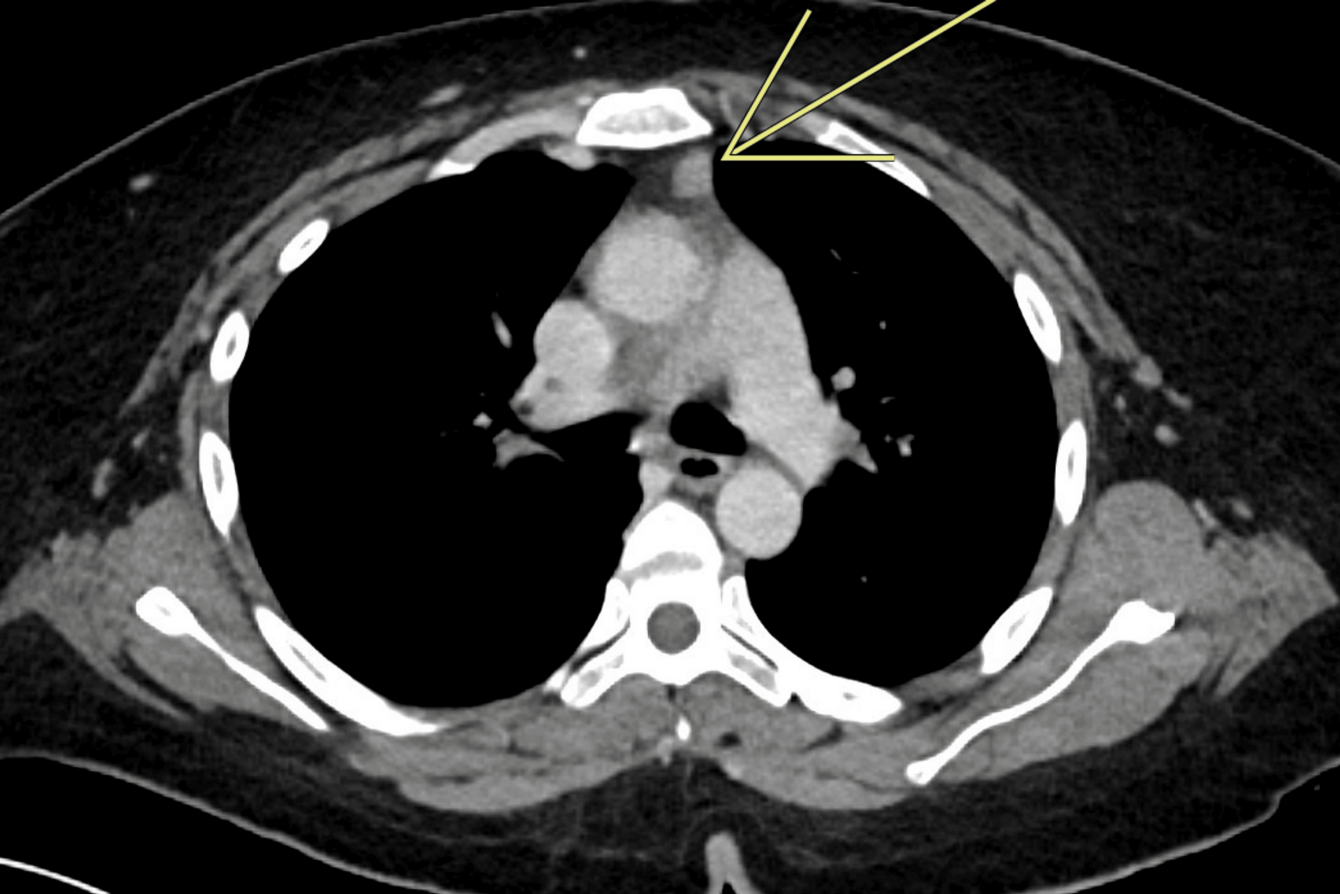
Chest computed tomography (CT) scan showed a small thymic hyperplasia (thymoma type B2, Masaoka-Koga stage 1)

A 45-year-old Saudi woman was diagnosed with generalized MG at a primary hospital one month prior to admission to our hospital and was treated with 60 mg of pyridostigmine and 15 mg of prednisolone. She was referred to our hospital and admitted for a myasthenic crisis. Upon admission, the patient presented with shortness of breath, dysphagia, dysarthria, ptosis in the right eye, and general muscle weakness. Computed tomography (CT) of the chest showed a small thymic hyperplasia (thymoma type B2, Masaoka-Koga stage 1; Figure 1).

The patient was admitted to the intensive care unit (ICU) for close monitoring and started on plasma exchange therapy along with IVIg. Given the severe symptoms and worsening condition, RTX therapy was initiated. RTX was administered as an intravenous infusion at a dose of 1000 mg every two weeks in the first cycle of treatment, followed by maintenance doses every six months [18].

**Figure 2:**
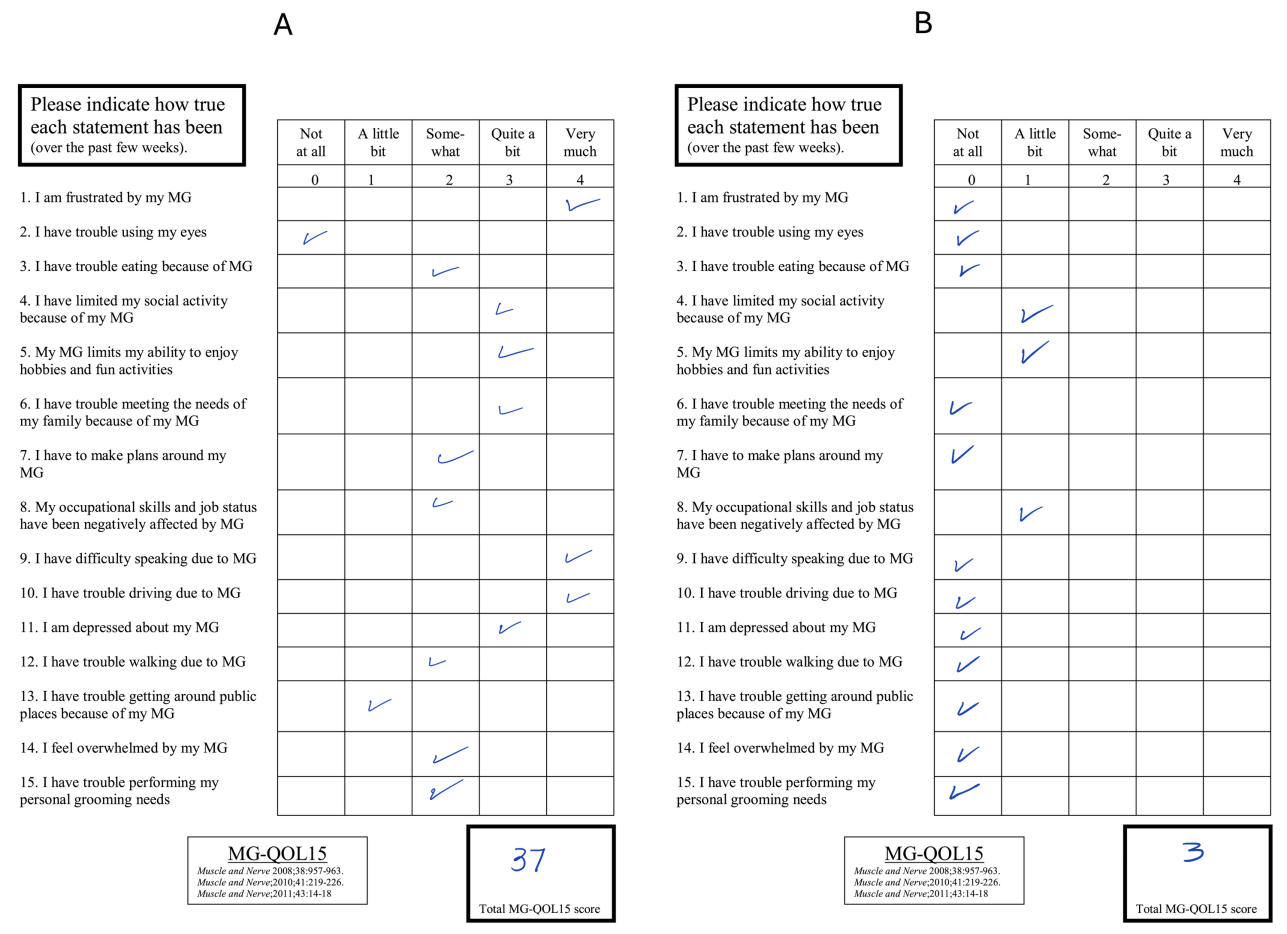
Contrast the MG-QOL-15 score of case one before (A) and after (B) receiving the RTX therapy with a four-month interval showing an improvement from 37 to 3 MG-QOL-15 - Myasthenia Gravis Quality of Life 15; RTX - rituximab

The patient showed significant improvement after the first cycle of RTX therapy, with reduced disease severity and improved muscle weakness. The patient's MG-QOL-15 score was assessed in the clinic four months after the first treatment, showing an improvement from 37 to 3 (Figure 2). The patient tolerated RTX therapy well and reported no adverse events. 

Case two

A 58-year-old Saudi woman with a history of follicular lymphoma with multiple involvements visited the emergency department with complaints of diplopia and worsening proximal muscle weakness three weeks prior. She had a history of dysphagia, dysarthria, shortness of breath, and a weak cough. Upon examination, the patient was conscious and oriented, with a breath count of 10 per minute, pupils reactive to light, bilateral ptosis, and limited horizontal extraocular movements. Additionally, the patient experienced fatigue during repeated muscle testing. She had received one cycle of a bendamustine chemotherapy regimen for lymphoma one year prior. As with other drugs, bendamustine has side effects such as constipation, diarrhea, nausea, vomiting, neutropenia, anemia, lymphopenia, etc [19]. Given the patient's history of lymphoma and worsening symptoms of MG, RTX therapy was initiated as an off-label use. 

**Figure 3:**
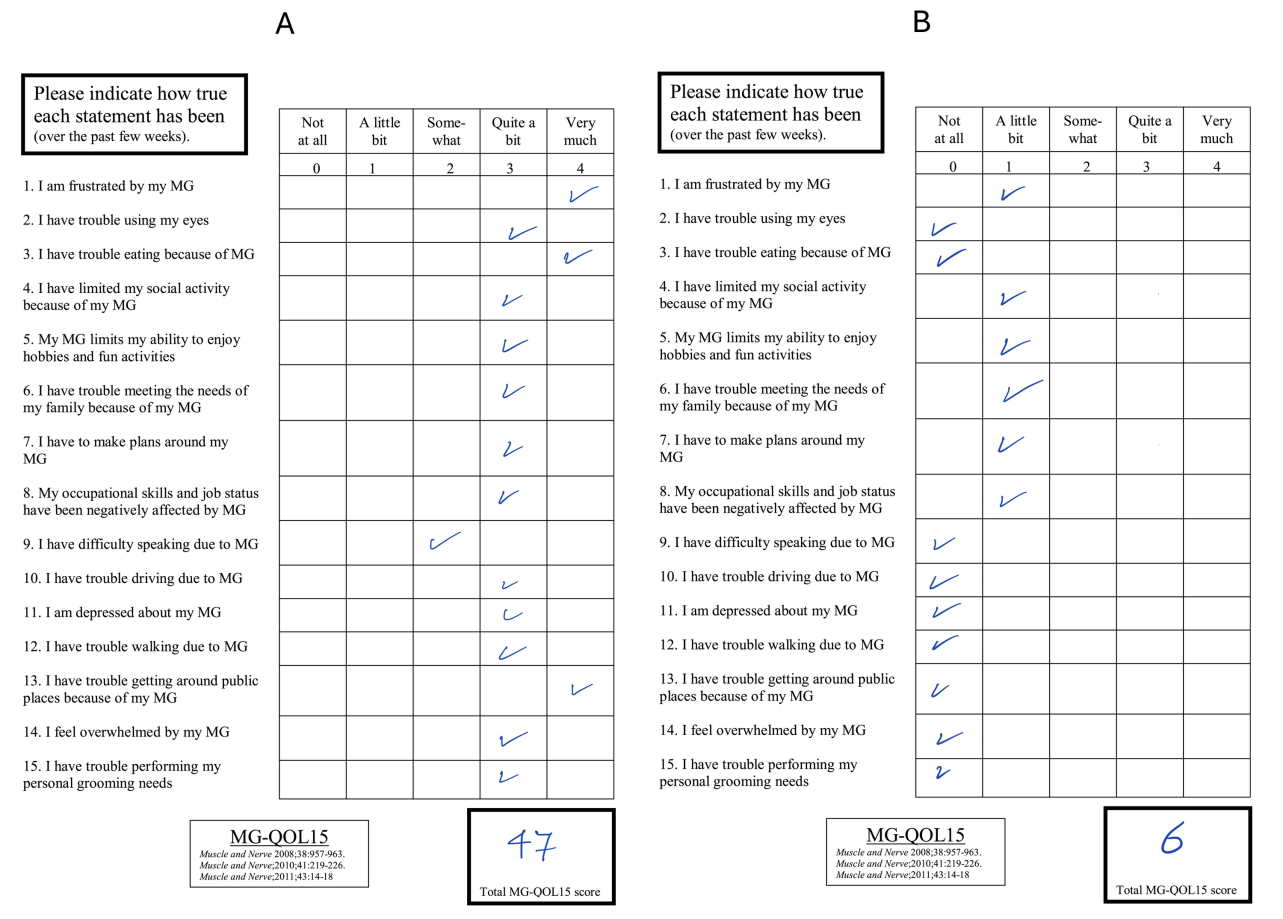
Contrast the MG-QOL-15 score of case two before (A) and after (B) receiving the RTX therapy with a four-month interval showing an improvement from 47 to 6 MG-QOL-15 - Myasthenia Gravis Quality of Life 15; RTX - rituximab

At the most recent follow-up, four months later, the patient showed significant improvements in muscle weakness and overall clinical condition after receiving RTX therapy. The patient's MG-QOL-15 score improved from 47 to 6 at the four-month follow-up visit (Figure 3).

Case three

**Figure 4:**
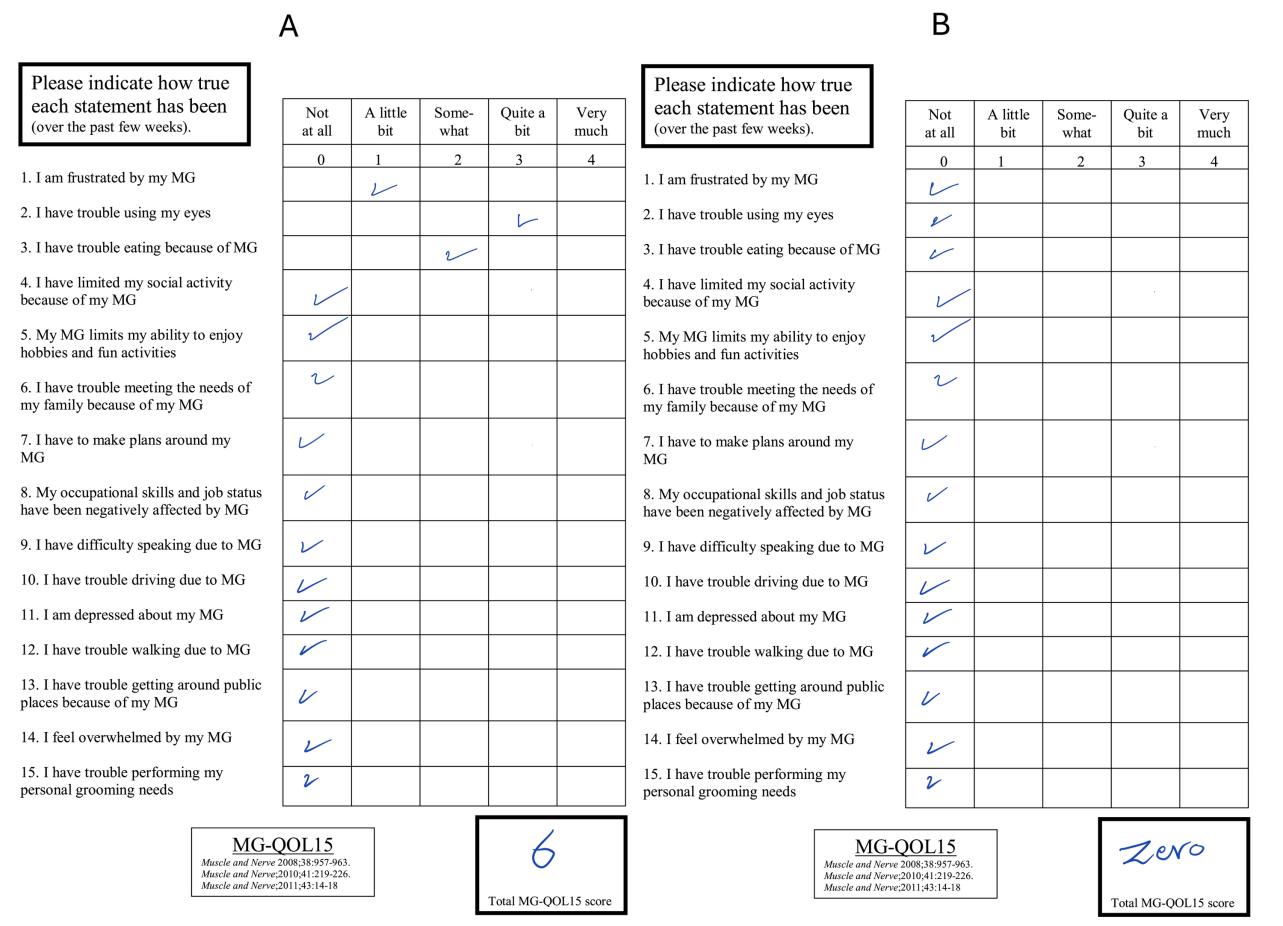
Contrast the MG-QOL-15 score of case three before (A) and after (B) receiving the RTX therapy with a four-month interval showing an improvement from 6 to 0 MG-QOL-15 - Myasthenia Gravis Quality of Life 15; RTX - rituximab

A 46-year-old Saudi man with a history of type 2 diabetes mellitus, mild dysphagia, hypertension, dyslipidemia, asthma, and ptosis was referred from an ophthalmology clinic to a neuromuscular clinic for the evaluation of progressive unilateral ptosis. On assessment, the patient was confirmed to have generalized MG and tested positive for AChR-Ab. Given the patient's history of generalized MG and positive test results for AChR-Ab, RTX therapy was initiated as a first-line non-corticosteroid immunosuppressive drug. During the first clinic visit four months later, the patient reported a significant improvement in his symptoms of ptosis. His MG-QOL-15 score improved from 6 to 0 at the four-month follow-up (Figure 4).

## Discussion

MG is the most prevalent neuromuscular junction disorder and involves several dysfunctions that eventually lead to muscle fatigability and weakness [1]. Treatment options for MG include various immunotherapeutics, such as oral immunosuppressants, which are often used to reduce the adverse effects of long-term corticosteroid use [20, 21]. However, the onset of the clinical effects of these drugs can take months [22]. Recently, the US Food and Drug Administration (FDA) approved novel agents, such as eculizumab, to treat AChR-associated generalized MG [23, 24]. However, treatment-refractory cases remain a challenge and may require the use of multiple immunosuppressants with no benefit. Therefore, identifying safe and effective therapies for MG is crucial. Several studies have shown that RTX therapy is a potentially effective treatment option for patients with MG [25-29]. 

In this retrospective analysis, three cases of new-onset non-refractory generalized MG were treated with RTX as the first-line immunosuppressant therapy. All three patients enrolled in this study were positive for AChR-Ab. Notably, most MG patients have serum AChR-Ab and, less frequently, antibodies that target lipoprotein receptor-related protein 4 or MuSK [30]. 

According to Gilhus et al. [31], 10-15% of patients with MG are seronegative for antibodies. Therefore, identifying a seronegative group is challenging. Several observational studies have used RTX to treat seronegative patients with MG [14, 32]. A study conducted in 2015 demonstrated an 85% improvement in minimal manifestation status as assessed using the Minimal Manifestation Status Scale [14]. Another study conducted in 2019 showed improvement in the minimal manifestation status of four seronegative patients with MG treated with RTX [33]. A recent study published in 2021 used RTX therapy to treat 20 seronegative patients with MG, resulting in improvements in the minimal manifestation status of 40% of the patients and approximately half of the patients with MG and AChR-Ab [34]. In our study, RTX resulted in a significant improvement in the MG-QOL-15 score of patients. After starting RTX treatment, we observed significant improvements in disease symptoms and QOL in all patients during the follow-up period.

In our study, RTX improved the MG-QOL-15 scores of all cases by almost 100%. This led to the stabilization of case one and allowed surgeons to perform thymectomy, as the patient had thymoma type B2, Masaoka-Koga stage 1, upon admission. In case two, RTX improved the patient's QOL by treating both MG and lymphoma. This reduced the risk of drug toxicity and costs. Case three involved a surgeon who experienced MG symptoms, including diplopia and ptosis. His condition improved after the administration of RTX, and he returned to work normally. We also managed to decrease the oral prednisolone dose in all patients to less than 15 mg within six months, and no negative effects were observed. However, prednisolone was not completely withdrawn to prevent disease relapse.

Notably, over a period of six months, RTX therapy resulted in significant improvements in muscle weakness, overall clinical condition, and QOL in all patients. In addition, no MG crises or RTX-related side effects were observed. This suggests that RTX therapy may be safe and effective in patients with MG who test positive for AChR-Ab. 

Our study has several limitations, including a small population size, lack of a control group, and a short follow-up period. In addition, QOL was assessed with a single instrument (MG-QOL-15). Larger sample sizes and longer follow-up periods are required to better evaluate the long-term outcomes of RTX therapy in patients with MG.

## Conclusions

This retrospective analysis suggests that RTX therapy may be an effective and safe treatment option for patients with MG who test positive for AChR-Ab. For the first time in Saudi Arabia, we have used RTX as a first-line non-corticosteroid immunosuppressive therapy to treat three non-refractory MG cases. Our result shows that RTX therapy resulted in significant improvements in muscle weakness, overall clinical condition, and QOL in the three patients described in this analysis. Further research with larger patient populations and longer follow-up durations is warranted to confirm these findings and to better characterize the role of RTX in the management of MG.
